# Globally Optimal Distributed Kalman Filtering for Multisensor Systems with Unknown Inputs

**DOI:** 10.3390/s18092976

**Published:** 2018-09-06

**Authors:** Yali Ruan, Yingting Luo, Yunmin Zhu

**Affiliations:** College of Mathematics, Sichuan University, Chengdu 610064, Sichuan, China; ruanyali2018@163.com (Y.R.); ymzhu@scu.edu.cn (Y.Z.)

**Keywords:** optimal estimate, unknown inputs, distributed fusion, augmented state Kalman filtering (ASKF)

## Abstract

In this paper, the state estimation for dynamic system with unknown inputs modeled as an autoregressive AR (1) process is considered. We propose an optimal algorithm in mean square error sense by using difference method to eliminate the unknown inputs. Moreover, we consider the state estimation for multisensor dynamic systems with unknown inputs. It is proved that the distributed fused state estimate is equivalent to the centralized Kalman filtering using all sensor measurement; therefore, it achieves the best performance. The computation complexity of the traditional augmented state algorithm increases with the augmented state dimension. While, the new algorithm shows good performance with much less computations compared to that of the traditional augmented state algorithms. Moreover, numerical examples show that the performances of the traditional algorithms greatly depend on the initial value of the unknown inputs, if the estimation of initial value of the unknown input is largely biased, the performances of the traditional algorithms become quite worse. However, the new algorithm still works well because it is independent of the initial value of the unknown input.

## 1. Introduction

The classic Kalman filtering (KF) [1] requires the model of the dynamic system is accurate. However, in many realistic situations, the model may contain unknown inputs in process or measurement equations. The issue concerning estimating the state of a linear time-varying discrete time system with unknown inputs is widely studied by researchers. One widely adopted approach is to consider the unknown inputs as part of the system state and then estimate both of them. This leads to an augmented state Kalman filtering (ASKF). Its computational cost increases due to the augmented state dimension. It is proposed by Friedland [2] in 1969 a two-stage Kalman filtering (TSKF) to reduce the computation complexity of the ASKF, which is optimal for the situation of a constant unknown input. On the basis of the work in [2], it is proposed by Hsieh et al. an optimal two-stage algorithm (OTSKF) for the dynamic system with random bias and a robust two-stage algorithm for the dynamic system with unknown inputs in 1999 [3] and 2000 [4] respectively. It is assumed in [3,4,5] that the unknown inputs were an autoregressive AR (1) process, with the two-stage algorithms being optimal in the mean square error (MSE) sense. However, the optimality of the ASKF and OTSKF depends on the premise that the initial value of the unknown measurement can be estimated correctly. Under the condition of incorrect initial value of the unknown measurement, the ASKF and OTSKF will have poor performance (see Examples 1 and 2 in Section 5), especially, when the unknown measurement is not stationary as regarded in [4,5]. Due to the difficulty of knowing the exact initial value of the unknown measurement, improvements should be made on these approaches. Many other researchers have focused on the problem of unknown inputs [6,7,8] in recent years.

A large number of sensors are now used in practical applications in numerous advanced systems. With the processing center receiving all measurements from the local sensors in time, centralized Kalman filtering (CKF) can be accomplished, and the resulting state estimates are optimal in the MSE. Nevertheless, because of limited communication bandwidth, and relatively low survivability of the system in unfavorable conditions, like martial circumstances, Kalman filtering is required to be carried on every local sensor upon its own observation first for local requirement, and then the processed data-local state estimate is transmitted to a fusion center. Therefore, the fusion center now needs to fuse all the local estimates received to produce a globally optimal or suboptimal state estimate. In the existing research literatures, a large number of researches on distributed Kalman filtering (DKF) have been done. Under certain common conditions, particularly, the supposition of cross-independent sensor noises, an optimal DKF fusion was proposed in [9,10,11] respectively, which was proved to be the same as the CKF adopting all sensor measurements, illustrating that it is universally optimal. Besides, a Kalman filtering fusion with feedback was also suggested there. Then, it was presented in [12] a rigorous performance analysis for Kalman filtering fusion with feedback. The results mentioned above are effective only for conditions with uncoupled observation noises across sensors. It is demonstrated by Song et al. [13] that when the sensor noises are cross-correlated, the fused state estimate was also equal to the CKF under a mild condition. Similarly with [13], Luo et al. [14] posed a distributed Kalman filtering fusion with random state transition and measurement matrices, i.e., random parameter matrices Kalman filtering in 2008. Moreover, they proved that under a mild condition the distributed fusion state estimate is equivalent to the centralized random parameter matrices Kalman filtering using all sensor measurement, which under the assumption that the expectation of all sensor measurement matrices are of full row rank. As far as we know, few studies have been done for multisensor system with unknown inputs by the above mentioned augmented methods. The main reason is that the augmented methods greatly increase the state dimension and computation complexity for the multisensor system.

In this paper, an optimal estimation for dynamic system with unknown inputs in the MSE sense is proposed. Different from the work in [2,3,4,5], the approach of eliminating instead of estimating the unknown inputs is used. The unknown inputs are assumed to be an autoregressive AR (1) process and are eliminated by measurement difference method. Then the original dynamic system is converted to a remodeled system with correlated process and measurement noises. The new measurement noise of the remodeled system in this paper is not only one-step correlated in time but also correlated with the process noise. We propose a globally optimal recursive state estimate algorithm for this remodeled system. Compared with the ASKF and OTSKF, the new algorithm is still optimal in the MSE sense but with less computation stress. Additionally, it is showed that the performance of the new algorithm does not rely on the initial value of the unknown input. For the multisensor system with unknown inputs, we show that the centralized filtering can still be expressed by a linear combination of the local estimates. Therefore, the performance of the distributed filtering fusion is the same as that of the centralized fusion. The new algorithm is optimal in the MSE sense with low computation complexity. Numerical examples are given to support our analysis.

The remainder of this paper is organized as follows: the problem formulation is discussed in Section 2, followed by an optimal estimation algorithm for dynamic system with unknown inputs being put forward in Section 3. In Section 4, a distributed algorithm for multisensor system with unknown inputs will be given, demonstrating that the fused state estimate is equal to the centralized Kalman filtering with all sensor measurements. Several simulation examples are given in Section 5. Section 6 is the summary of our analysis and possible future work.

## 2. Problem Formulation

Consider a discrete time dynamic system:

(1) $$x_{k+1}=F_{k}x_{k}+\nu_{k},$$

(2) $$y_{k}=H_{k}x_{k}+A_{k}d_{k}+\omega_{k},$$

where $x_{k}\in R^{m}$ is the system state, $\nu_{k}\in R^{m}$ is the measurement vector, the process noise and measurement noise $\omega_{k}\in R^{n}$ are zero-mean white noise sequences with the following covariances:

(3) $$E(\nu_{k}\nu_{j}^{T})=R_{\nu_{k}}\delta_{k-j},$$

(4) $$E(\omega_{k}\omega_{j}^{T})=R_{\omega_{k}}\delta_{k-j},$$

(5) $$E(\nu_{k}\omega_{j}^{T})=0,\;\forall k,j.$$

where:

$$\delta_{k-j}=\left\{ \begin{array}{l} 1,\;k=j\\ 0,\;k\neq j \end{array}\right.,$$

$d_{k}\in R^{p}$ is the unknown input. Matrices $F_{k},H_{k}$ and $A_{k}$ are of appropriate dimensions by assuming that $A_{k}\in R^{n\times p}$ is of full column rank, i.e., $rank(A_{k})=p$. Therefore, we have ${\left(A_{k}\right)}^{†}A_{k}=I$, where the superscript “†” denotes Moore-Penrose pseudo inverse. It is assumed $d_{k}$ follows an autoregressive AR (1), i.e.,:

(6) $$d_{k+1}=B_{k}d_{k}+\omega_{d_{k}},$$

where $B_{k}$ is nonsingular and $\omega_{d_{k}}$ is a zero-mean white noise sequences with covariance:

(7) $$E(\omega_{d_{k}}\omega_{d_{j}}^{T})=R_{d_{k}}\delta_{k-j}.$$

This model is widely considered in [2,3,4,5]. For example, in radar systems, the measurement often contains a fixed unknown deviation or an unknown deviation that gradually increases as the distance becomes longer. Such deviations can be described by Equation (6).

ASKF and OTSKF are two classic algorithms to handle this problem. The ASKF regards $x_{k}$ and $d_{k}$ as an augmented state and estimates them together, while the OTSKF estimates $x_{k}$ and $d_{k}$ respectively at first and then fusions them to achieve the optimal estimation. As a matter of fact, the unknown inputs can be eliminated easily by difference method. Denote:

(8) $$z_{k}=B_{k}^{-1}A_{k+1}^{†}y_{k+1}-A_{k}^{†}y_{k}.$$

Equations (1) and (2) can be represented as:

(9) $$x_{k+1}=F_{k}x_{k}+\nu_{k},$$

(10) $$z_{k}=M_{k}x_{k}+u_{k},$$

where:

(11) $$M_{k}=B_{k}^{-1}A_{k+1}^{†}H_{k+1}F_{k}-A_{k}^{†}H_{k},$$

(12) $$u_{k}=B_{k}^{-1}A_{k+1}^{†}H_{k+1}\nu_{k}+B_{k}^{-1}\omega_{d_{k}}+B_{k}^{-1}A_{k+1}^{†}\omega_{k+1}-A_{k}^{†}\omega_{k}.$$

From Equation (12), it is not difficult to find out that the new measurement noise $u_{k}$ is one-step correlated and correlates with the process noise, i.e.,:

(13) $$E(u_{k}\nu_{j}^{T})=B_{k}^{-1}A_{k+1}^{†}H_{k+1}R_{\nu_{k}}\delta_{k-j},$$

(14) $$\begin{array}{ll} E(u_{k}u_{k}^{T}) & =B_{k}^{-1}A_{k+1}^{†}H_{k+1}R_{\nu_{k}}H_{k+1}^{T}{\left(A_{k+1}^{†}\right)}^{T}{\left(B_{k}^{-1}\right)}^{T}+B_{k}^{-1}R_{d_{k}}{\left(B_{k}^{-1}\right)}^{T}\\ & +B_{k}^{-1}A_{k+1}^{†}R_{\omega_{k+1}}{\left(A_{k+1}^{†}\right)}^{T}{\left(B_{k}^{-1}\right)}^{T}+A_{k}^{†}R_{\omega_{k}}{\left(A_{k}^{†}\right)}^{T}, \end{array}$$

(15) $$E(u_{k}u_{j}^{T})=-A_{k}^{†}R_{\omega_{k}}{\left(A_{k}^{†}\right)}^{T}{\left(B_{k-1}^{-1}\right)}^{T}\delta_{k-j+1},k\neq j.$$

## 3. Optimal Estimation for the Remodeled System 

It is assumed in the classic Kalman filtering that the process noises and measurement noises are uncorrelated temporally; in the meantime, both noises are mutually uncorrelated except at the same time instant. The noises in Equations (13)–(15) apparently violate these assumptions. Using the latest research achievements about Kalman filtering with correlated noises in [15,16,17,18,19,20], we can give an optimal estimation for the remodeled system (9) and (10) in the MSE sense. The recursive state estimate of the new system is presented in the following theorem.

**Theorem** **1.** *The globally optimal estimate for the remodeled system (9) and (10) is given by:*

$$x_{k|k}=x_{k|k-1}+J_{k}L_{k}^{†}\Delta z_{k},$$

$$P_{k|k}=P_{k|k-1}-J_{k}L_{k}^{†}J_{k}^{T},$$

*where:*

(16) $$x_{k|k-1}=F_{k-1}x_{k-1|k-1}+R_{\nu_{k-1}}H_{k}^{T}{\left(A_{k}^{†}\right)}^{T}{\left(B_{k-1}^{-1}\right)}^{T}L_{k-1}^{†}\Delta z_{k-1},$$

(17) $$\begin{array}{cc} P_{k|k-1}= & E(x_{k}-x_{k|k-1}){\left(x_{k}-x_{k|k-1}\right)}^{T}\\ = & F_{k-1}P_{k-1|k-1}F_{k-1}^{T}-F_{k-1}J_{k-1}L_{k-1}^{†}B_{k-1}^{-1}A_{k}^{†}H_{k}R_{\nu_{k-1}}\\ & -R_{\nu_{k-1}}H_{k}^{T}{\left(A_{k}^{†}\right)}^{T}{\left(B_{k-1}^{-1}\right)}^{T}L_{k-1}^{†}J_{k-1}^{T}F_{k-1}^{T}+R_{\nu_{k-1}}\\ & -R_{\nu_{k-1}}H_{k}^{T}{\left(A_{k}^{†}\right)}^{T}{\left(B_{k-1}^{-1}\right)}^{T}L_{k-1}^{†}B_{k-1}^{-1}A_{k}^{†}H_{k}R_{\nu_{k-1}} \end{array}$$

(18) $$\begin{array}{cc} \Delta z_{k} & =z_{k}-z_{k|k-1},\\ & =z_{k}-M_{k}x_{k|k-1}+A_{k}^{†}R_{\omega_{k}}{\left(A_{k}^{†}\right)}^{T}{\left(B_{k-1}^{-1}\right)}^{T}L_{k-1}^{†}\Delta z_{k-1}, \end{array}$$

(19) $$\begin{array}{cc} J_{k}= & E(x_{k}-x_{k|k-1}){\left(z_{k}-z_{k|k-1}\right)}^{T},\\ = & P_{k|k-1}M_{k}^{T}+(F_{k-1}J_{k-1}L_{k-1}^{†}+R_{\nu_{k-1}}H_{k}^{T}{\left(A_{k}^{†}\right)}^{T}{\left(B_{k-1}^{-1}\right)}^{T}L_{k-1}^{†})\\ & \cdot B_{k-1}^{-1}A_{k}^{†}R_{\omega_{k}}{\left(A_{k}^{†}\right)}^{T}, \end{array}$$

(20) $$\begin{array}{cc} L_{k}= & E(z_{k}-z_{k|k-1}){\left(z_{k}-z_{k|k-1}\right)}^{T},\\ = & M_{k}J_{k}+A_{k}^{†}R_{\omega_{k}}{\left(A_{k}^{†}\right)}^{T}{\left(B_{k-1}^{-1}\right)}^{T}L_{k-1}^{†}(J_{k-1}^{T}F_{k-1}^{T}+B_{k-1}^{-1}A_{k}^{†}H_{k}R_{\nu_{k-1}})\\ & \cdot M_{k}^{T}+R_{u_{k}}-A_{k}^{†}R_{\omega_{k}}{\left(A_{k}^{†}\right)}^{T}{\left(B_{k-1}^{-1}\right)}^{T}L_{k-1}^{†}B_{k-1}^{-1}A_{k}^{†}R_{\omega_{k}}{\left(A_{k}^{†}\right)}^{T}. \end{array}$$

**Remark** **1.** *From Theorem 1, the new algorithm presented in this section is optimal in the MSE sense. In theory, the ASKF and OTSKF are also optimal in the MSE sense (see [2,3]). Nevertheless, the optimality of the ASKF and OTSKF depends on the assumption that the initial condition of the unknown measurement* $d_{0|0}=E(d_{0})$ *, which is difficult to meet in real situations. It will be demonstrated by numerical examples in the Section 5 that if the initial value of the unknown input is wrong, their performances will be greatly influenced. By contrast, the new algorithm will continue its good performance as it does not rely on the initial value of the unknown input.*

**Remark** **2.** *A flop is defined as one addition, subtraction and multiplication. To estimate the complexity of an algorithm, the total number of flops is counted, expressing it as a polynomial of the dimensions of the matrices and vectors involved, and simplifying the expression by ignoring all terms except the leading terms. Then the complexities of the ASKF, OTSKF and the new algorithm are equivalent to* $O(m^{3}+n^{3}+p^{3}+m^{2}n+mn^{2}+m^{2}p+mp^{2}+n^{2}p+np^{2}+mnp)$ *. The evaluation complexities of the three algorithms are the same order polynomials. We will compare their complexities more precisely by numerical examples in Section 5.*

## 4. Multisensor Fusion

The l-sensor dynamic system is given by:

$$x_{k+1}=F_{k}x_{k}+\nu_{k},\;k=0,1,\ldots$$

(21) $$y_{k}^{i}=H_{k}^{i}x_{k}+A_{k}^{i}d_{k}^{i}+\omega_{k}^{i},$$

$$d_{k+1}^{i}=B_{k}^{i}d_{k}^{i}+\omega_{d_{k}}^{i},\;i=1,\ldots ,l$$

where $x_{k}\in R^{m}$ is the system state, $y_{k}^{i}\in R^{n_{i}}$ is the measurement vector in the i-th sensor, $\nu_{k}\in R^{m}$ is the process noise and $\omega_{k}^{i}\in R^{n_{i}}$ is measurement noise, $d_{k}^{i}\in R^{p_{i}}$ is the unknown input in i-th sensor. Matrices $F_{k},H_{k}^{i}$ and $A_{k}^{i}$ are of appropriate dimensions. 

We assume the system has the following statistical properties:

(1) Every single sensor satisfies the assumption in Section 2.
(2) $A_{k}^{i}\in R^{n_{i}\times p_{i}}$ is of full column rank, then ${\left(A_{k}^{i}\right)}^{†}A_{k}^{i}=I$.
(3) $\{\nu_{k},\omega_{k}^{j},k=0,1,2,\ldots \},i,j=1,\ldots ,l$ is a sequence of independent variables.

Similarly to Equations (9) and (10), Equation (21) could be converted to:

(22) $$x_{k+1}=F_{k}x_{k}+\nu_{k},\;k=0,1,\ldots$$

(23) $$z_{k}^{i}=M_{k}^{i}x_{k}+u_{k}^{i},\;i=1,\ldots ,l$$

where:

$$M_{k}^{i}=B_{k}^{i^{-1}}A_{k+1}^{i^{†}}H_{k+1}^{i}F_{k}-A_{k}^{i^{†}}H_{k}^{i},$$

$$u_{k}^{i}=B_{k}^{i^{-1}}A_{k+1}^{i^{†}}H_{k+1}^{i}\nu_{k}+B_{k}^{i^{-1}}\omega_{d_{k}}^{i}+B_{k}^{i^{-1}}A_{k+1}^{i^{†}}\omega_{k+1}^{i}-A_{k}^{i^{†}}\omega_{k}^{i}.$$

The stacked measurement equation is written as:

$$z_{k}=M_{k}x_{k}+u_{k}$$

where:

$$z_{k}={\left(z_{k}^{1^{T}},\ldots ,z_{k}^{l^{T}}\right)}^{T},M_{k}={\left(M_{k}^{1^{T}},\ldots ,M_{k}^{l^{T}}\right)}^{T},u_{k}={\left(u_{k}^{1^{T}},\ldots ,u_{k}^{l^{T}}\right)}^{T}.$$

According to Theorem 1, the local Kalman filtering at the i-th sensor is:

(24) $$x_{k|k}^{i}=x_{k|k-1}^{i}+J_{k}^{i}L_{k}^{i^{†}}\Delta z_{k}^{i},$$

(25) $$x_{k|k-1}^{i}=F_{k-1}x_{k-1|k-1}^{i}+R_{\nu_{k-1}}H_{k}^{i^{T}}{\left(A_{k}^{i^{†}}\right)}^{T}{\left(B_{k-1}^{i^{-1}}\right)}^{T}L_{k-1}^{i^{†}}\Delta z_{k-1}^{i},$$

with covariances of filtering error given by:

$$P_{k|k}^{i}=P_{k|k-1}^{i}-J_{k}^{i}L_{k}^{i^{†}}J_{k}^{i^{T}},$$

where:

(26) $$\Delta z_{k}^{i}=z_{k}^{i}-M_{k}^{i}x_{k|k-1}^{i}+A_{k}^{i^{†}}R_{\omega_{k}}^{i}{\left(A_{k}^{i^{†}}\right)}^{T}{\left(B_{k-1}^{i^{-1}}\right)}^{T}L_{k-1}^{i^{†}}\Delta z_{k-1}^{i},$$

$$J_{k}^{i}=E(x_{k}^{i}-x_{k|k-1}^{i}){\left(z_{k}^{i}-z_{k|k-1}^{i}\right)}^{T},$$

$$L_{k}^{i}=E(z_{k}^{i}-z_{k|k-1}^{i}){\left(z_{k}^{i}-z_{k|k-1}^{i}\right)}^{T},$$

$$P_{k|k-1}^{i}=E(x_{k}^{i}-x_{k|k-1}^{i}){\left(x_{k}^{i}-x_{k|k-1}^{i}\right)}^{T}.$$

According to Theorem 1, the centralized Kalman filtering with all sensor data is given by:

(27) $$x_{k|k}=x_{k|k-1}+J_{k}L_{k}^{†}\Delta z_{k},$$

(28) $$x_{k|k-1}=F_{k-1}x_{k-1|k-1}+R_{\nu_{k-1}}H_{k}^{T}{\left(A_{k}^{†}\right)}^{T}{\left(B_{k-1}^{-1}\right)}^{T}L_{k-1}^{†}\Delta z_{k-1},$$

The covariance of filtering error given by:

$$P_{k|k}=P_{k|k-1}-J_{k}L_{k}^{†}J_{k}^{T},$$

where:

$$A_{k}=diag(A_{k}^{1},\ldots ,A_{k}^{l}),B_{k}=diag(B_{k}^{1},\ldots ,B_{k}^{l}),$$

(29) $$\Delta z_{k}=z_{k}-M_{k}x_{k|k-1}+A_{k}^{†}R_{\omega_{k}}{\left(A_{k}^{†}\right)}^{T}{\left(B_{k-1}^{-1}\right)}^{T}L_{k-1}^{†}\Delta z_{k-1},$$

$$J_{k}=E(x_{k}-x_{k|k-1}){\left(z_{k}-z_{k|k-1}\right)}^{T},$$

$$L_{k}=E(z_{k}-z_{k|k-1}){\left(z_{k}-z_{k|k-1}\right)}^{T},$$

$$P_{k|k-1}=E(x_{k}-x_{k|k-1}){\left(x_{k}-x_{k|k-1}\right)}^{T}.$$

diag is the diagonalization of a matrix.

**Remark** **3.** *There are two key points to express the centralized filtering Equations (27) and (28) in terms of the local filtering:*

*(1) Taking into consideration the measurement noise of single sensor in new system Equations (22) and (23), it can be observed that the sensor noises of the converted system are cross-correlated even if the original sensor noises are mutually independent.*

*(2)* $\Delta z_{k}$ *in Equation (27) is not stacked by local* $\Delta z_{k}^{i}$ *in Equation (26) directly and includes* $\Delta z_{k-1}$ *in its expression, which makes our problem more complicated than the previous distributed problem in [9,10,11,12,13,14,21].*

Next, we will solve these two problems to express the centralized filtering Equation (28) in terms of the local filtering. We assume that $H_{k}^{T}$ is of full column rank. Thus, we have ${\left(H_{k}^{T}\right)}^{†}H_{k}^{T}=I$. Using (28), we can get:

(30) $$\Delta z_{k-1}=L_{k-1}{\left[{\left(B_{k-1}^{-1}\right)}^{T}\right]}^{-1}{\left[{\left(A_{k}^{†}\right)}^{T}\right]}^{†}{\left(H_{k}^{T}\right)}^{†}R_{\nu_{k-1}}^{-1}(x_{k|k-1}-F_{k-1}x_{k-1|k-1}).$$

Substituting (29) and (30) into (27), we have:

(31) $$\begin{array}{cc} x_{k|k}= & x_{k|k-1}+J_{k}L_{k}^{†}\Delta z_{k}\\ = & x_{k|k-1}+J_{k}L_{k}^{†}(z_{k}-M_{k}x_{k|k-1}+A_{k}^{†}R_{\omega_{k}}{\left(A_{k}^{†}\right)}^{T}{\left(B_{k-1}^{-1}\right)}^{T}L_{k-1}^{†}\Delta z_{k-1})\\ = & x_{k|k-1}+J_{k}L_{k}^{†}{\left(z_{k}-M_{k}x_{k|k-1}+A_{k}^{†}R_{\omega_{k}}{\left(A_{k}^{†}\right)}^{T}{\left(B_{k-1}^{-1}\right)}^{T}L_{k-1}^{†}L_{k-1}({\left(B_{k-1}^{-1}\right)}^{T})\right)}^{-1}{\left({\left(A_{k}^{†}\right)}^{T}\right)}^{†}\\ & \cdot {\left(H_{k}^{T}\right)}^{†}R_{\nu_{k-1}}^{-1}(x_{k|k-1}-F_{k-1}x_{k-1|k-1}))\\ = & x_{k|k-1}+J_{k}L_{k}^{†}(z_{k}-M_{k}x_{k|k-1}+A_{k}^{†}R_{\omega_{k}}{\left(H_{k}^{T}\right)}^{†}R_{\nu_{k-1}}^{-1}(x_{k|k-1}-F_{k-1}x_{k-1|k-1}))\\ = & x_{k|k-1}-J_{k}L_{k}^{†}M_{k}x_{k|k-1}+J_{k}L_{k}^{†}A_{k}^{†}R_{\omega_{k}}{\left(H_{k}^{T}\right)}^{†}R_{\nu_{k-1}}^{-1}(x_{k|k-1}-F_{k-1}x_{k-1|k-1})+J_{k}L_{k}^{†}z_{k}. \end{array}$$

Using (26), we have:

(32) $$z_{k}^{i}=\Delta z_{k}^{i}+M_{k}^{i}x_{k|k-1}^{i}-A_{k}^{i^{†}}R_{\omega_{k}}^{i}{\left(A_{k}^{i^{†}}\right)}^{T}{\left(B_{k-1}^{i^{-1}}\right)}^{T}L_{k-1}^{i^{†}}\Delta z_{k-1}^{i}.$$

We assume that $J_{k}^{i}\in R^{m\times n_{i}}$ is of full column rank, i.e., $rankJ_{k}^{i}=n_{i}$. Thus, we have ${\left(J_{k}^{i}\right)}^{†}J_{k}^{i}=I$. Then, using (24), we can get:

(33) $$\Delta z_{k}^{i}=L_{k}^{i}J_{k}^{i^{†}}(x_{k|k}^{i}-x_{k|k-1}^{i}).$$

To express the centralized filtering $x_{k|k}$ in terms of the local filtering, by (25), (32) and (33), we have:

(34) $$\begin{array}{cc} J_{k}L_{k}^{†}z_{k}= & J_{k}\sum_{i=1}^{l}L_{k}^{†}(*i)z_{k}^{i}\\ = & J_{k}\sum_{i=1}^{l}L_{k}^{†}(*i)(\Delta z_{k}^{i}+M_{k}^{i}x_{k|k-1}^{i}-A_{k}^{i^{†}}R_{\omega_{k}}^{i}{\left(A_{k}^{i^{†}}\right)}^{T}{\left(B_{k-1}^{i^{-1}}\right)}^{T}L_{k-1}^{i^{†}}\Delta z_{k-1}^{i})\\ = & J_{k}\sum_{i=1}^{l}L_{k}^{†}(*i)(L_{k}^{i}J_{k}^{i^{†}}(x_{k|k}^{i}-x_{k|k-1}^{i})+M_{k}^{i}x_{k|k-1}^{i}-A_{k}^{i^{†}}R_{\omega_{k}}^{i}{\left(A_{k}^{i^{†}}\right)}^{T}{\left(B_{k-1}^{i^{-1}}\right)}^{T}L_{k-1}^{i^{†}}\\ & \cdot L_{k-1}^{i}{\left({\left(B_{k-1}^{i^{-1}}\right)}^{T}\right)}^{-1}{\left({\left(A_{k}^{i^{†}}\right)}^{T}\right)}^{†}{\left(H_{k}^{i^{T}}\right)}^{†}R_{\nu_{k-1}}^{-1}(x_{k|k-1}^{i}-F_{k-1}x_{k-1|k-1}^{i}))\\ = & J_{k}\sum_{i=1}^{l}L_{k}^{†}(*i)(L_{k}^{i}J_{k}^{i^{†}}(x_{k|k}^{i}-x_{k|k-1}^{i})+M_{k}^{i}x_{k|k-1}^{i}-A_{k}^{i^{†}}R_{\omega_{k}}^{i}{\left(H_{k}^{i^{T}}\right)}^{†}R_{\nu_{k-1}}^{-1}\\ & \cdot (x_{k|k-1}^{i}-F_{k-1}x_{k-1|k-1}^{i})), \end{array}$$

where $L_{k}^{†}(*i)$ is the *i*-th column block of $L_{k}^{†}$.

Thus, substituting (34) into (31) yields:

(35) $$\begin{array}{cc} x_{k|k}= & x_{k|k-1}-J_{k}L_{k}^{†}M_{k}x_{k|k-1}+J_{k}L_{k}^{†}A_{k}^{†}R_{\omega_{k}}{\left(H_{k}^{†}\right)}^{T}R_{\nu_{k-1}}^{-1}(x_{k|k-1}-F_{k-1}x_{k-1|k-1})\\ & +J_{k}\sum_{i=1}^{l}L_{k}^{†}(*i)(L_{k}^{i}J_{k}^{i^{†}}(x_{k|k}^{i}-x_{k|k-1}^{i})+M_{k}^{i}x_{k|k-1}^{i}-A_{k}^{i^{†}}R_{\omega_{k}}^{i}{\left(H_{k}^{i^{T}}\right)}^{†}R_{\nu_{k-1}}^{-1}\\ & \cdot (x_{k|k-1}^{i}-F_{k-1}x_{k-1|k-1}^{i}))\\ = & (I-J_{k}L_{k}^{†}M_{k}+J_{k}L_{k}^{†}A_{k}^{†}R_{\omega_{k}}{\left(H_{k}^{†}\right)}^{T}R_{\nu_{k-1}}^{-1})x_{k|k-1}-J_{k}L_{k}^{†}A_{k}^{†}R_{\omega_{k}}{\left(H_{k}^{†}\right)}^{T}R_{\nu_{k-1}}^{-1}F_{k-1}x_{k-1|k-1}\\ & +J_{k}\sum_{i=1}^{l}L_{k}^{†}(*i)(L_{k}^{i}J_{k}^{i^{†}}x_{k|k}^{i}+(M^{i}-L_{k}^{i}J_{k}^{i^{†}}-A_{k}^{i^{†}}R_{\omega_{k}}^{i}{\left(H_{k}^{i^{T}}\right)}^{†}R_{\nu_{k-1}}^{-1})x_{k|k-1}^{i}\\ & +A_{k}^{i^{†}}R_{\omega_{k}}^{i}{\left(H_{k}^{i^{T}}\right)}^{†}R_{\nu_{k-1}}^{-1}F_{k-1}x_{k-1|k-1}^{i}). \end{array}$$

Similarly to Equation (35), using Equations (24), (26), (29) and (32), we have:

(36) $$\begin{array}{cc} x_{k|k-1}= & F_{k-1}x_{k-1|k-1}-R_{\nu_{k-1}}H_{k}^{T}{\left(A_{k}^{†}\right)}^{T}{\left(B_{k-1}^{-1}\right)}^{T}L_{k-1}^{†}(M_{k-1}x_{k-1|k-2}-A_{k-1}^{†}R_{\omega_{k-1}}\\ & \cdot {\left(H_{k-1}^{T}\right)}^{†}R_{\nu_{k-2}}^{-1}(x_{k-1|k-2}-F_{k-2}x_{k-2|k-2}))+R_{\nu_{k-1}}H_{k}^{T}{\left(A_{k}^{†}\right)}^{T}{\left(B_{k-1}^{-1}\right)}^{T}\\ & \cdot \sum_{i=1}^{l}L_{k-1}^{†}(*i)(L_{k-1}^{i}{\left({\left(B_{k-1}^{i^{-1}}\right)}^{T}\right)}^{-1}{\left({\left(A_{k}^{i^{†}}\right)}^{T}\right)}^{†}{\left(H_{k}^{i^{T}}\right)}^{†}R_{\nu_{k-1}}^{-1}(x_{k|k-1}^{i}-F_{k-1}x_{k-1|k-1}^{i})\\ & +M_{k-1}^{i}x_{k-1|k-2}^{i}-A_{k-1}^{i^{†}}R_{\omega_{k-1}}^{i}{\left(H_{k-1}^{i^{T}}\right)}^{†}R_{\nu_{k-2}}^{-1}(x_{k-1|k-2}^{i}-F_{k-2}x_{k-2|k-2}^{i}))\\ = & F_{k-1}x_{k-1|k-1}-R_{\nu_{k-1}}H_{k}^{T}{\left(A_{k}^{†}\right)}^{T}{\left(B_{k-1}^{-1}\right)}^{T}L_{k-1}^{†}(M_{k-1}-A_{k-1}^{†}R_{\omega_{k-1}}{\left(H_{k-1}^{T}\right)}^{†}R_{\nu_{k-2}}^{-1})\\ & \cdot x_{k-1|k-2}-R_{\nu_{k-1}}H_{k}^{T}{\left(A_{k}^{†}\right)}^{T}{\left(B_{k-1}^{-1}\right)}^{T}L_{k-1}^{†}A_{k-1}^{†}R_{\omega_{k-1}}{\left(H_{k-1}^{T}\right)}^{†}R_{\nu_{k-2}}^{-1}F_{k-2}x_{k-2|k-2}\\ & +R_{\nu_{k-1}}H_{k}^{T}{\left(A_{k}^{†}\right)}^{T}{\left(B_{k-1}^{-1}\right)}^{T}\sum_{i=1}^{l}L_{k-1}^{†}(*i)(L_{k-1}^{i}{\left({\left(B_{k-1}^{i^{-1}}\right)}^{T}\right)}^{-1}{\left({\left(A_{k}^{i^{†}}\right)}^{T}\right)}^{†}{\left(H_{k}^{i^{T}}\right)}^{†}\\ & \cdot R_{\nu_{k-1}}^{-1}x_{k|k-1}^{i}-L_{k-1}^{i}{\left({\left(B_{k-1}^{i^{-1}}\right)}^{T}\right)}^{-1}{\left({\left(A_{k}^{i^{†}}\right)}^{T}\right)}^{†}{\left(H_{k}^{i^{T}}\right)}^{†}R_{\nu_{k-1}}^{-1}F_{k-1}x_{k-1|k-1}^{i}\\ & +(M_{k-1}^{i}-A_{k-1}^{i^{†}}R_{\omega_{k-1}}^{i}{\left(H_{k-1}^{i^{T}}\right)}^{†}R_{\nu_{k-2}}^{-1})x_{k-1|k-2}^{i}+A_{k-1}^{i^{†}}R_{\omega_{k-1}}^{i}\\ & \cdot {\left(H_{k-1}^{i^{T}}\right)}^{†}R_{\nu_{k-2}}^{-1}F_{k-2}x_{k-2|k-2}^{i}). \end{array}$$

That means the centralized filtering is expressed in terms of the local filtering. Therefore, the distributed fused state estimate is equal to the centralized Kalman filtering adopting all sensor measurements, which means the distributed fused state estimate achieves the best performance.

**Remark** **4.** *From this new algorithm, it is easy to see that local sensors should transmit* $x_{k|k}^{i}$ *,* $x_{k|k-1}^{i}$ *,* $P_{k|k}^{i}$ *and* $P_{k|k-1}^{i}$ *to the fusion center to get global fusion result. The augmented methods greatly increase the state dimension and computation complexity for the multisensor system. Since the difference method does not increase the state dimension, the computation complexity is lower than that of the augmented method for the multisensory system.*

## 5. Numerical Examples

In this section, several simulations will be carried out for dynamic system with unknown inputs. It is assumed that the unknown input $d_{k+1}=B_{k}d_{k}+\omega_{k}$ in this paper. Actually, the unknown measurement $d_{k}$ is a stationary time series if the eigenvalue of $B_{k}$ is less than 1, or else the unknown measurement $d_{k}$ is a non-stationary time series. The performances of the new algorithm (denoted as Difference KF) in these two cases are discussed in Example 1 and 2, respectively:

**Example** **1.** *A two dimension target tracking problem is considered. The target dynamic models are given as Equations (1)–(7). The state transition matrices:*

$$F_{k}=\left(\begin{array}{cccc} 1 & 1 & 0 & 0\\ 0 & 1 & 0 & 0\\ 0 & 0 & 1 & 1\\ 0 & 0 & 0 & 1 \end{array}\right)$$

*and the measurement matrix is given by:*

$$H_{k}=\left(\begin{array}{cccc} 1 & 0 & 0 & 0\\ 0 & 0 & 1 & 0 \end{array}\right)$$

*Suppose* $A_{k}$ *is an identity matrix with appropriate dimensions,* $B_{k}=0.9I$ *. In this case,* $d_{k}$ *is a stationary time series. The targets start at* $x_{0}={\left(50,1,50,1\right)}^{T}$ *and the initial value* $d_{0}={\left(5,5\right)}^{T}$ *. The covariance matrices of the noises are given by:*

$$R_{\nu_{k}}=\left(\begin{array}{cccc} 1 & 0 & 0 & 0\\ 0 & 0.1 & 0 & 0\\ 0 & 0 & 1 & 0\\ 0 & 0 & 0 & 0.1 \end{array}\right),$$

$$R_{\omega_{k}}=\left(\begin{array}{cc} 1 & 0\\ 0 & 1 \end{array}\right),\;R_{d_{k}}=\left(\begin{array}{cc} 1 & 0\\ 0 & 1 \end{array}\right).$$

In the following, the computer time and performances of the ASKF, OTSKF and Difference KF will be compared respectively.

- Computer time

The complexities of the three algorithms are analyzed in Remark 2, which shows the complexities of the three algorithms are the equivalent order polynomials. Now let us compare their computer time by this example. Table 1 illustrates the computer time of the three algorithms with 1000 Monte-Carlo runs respectively, through which we can find out that the new algorithm is the fastest algorithm in this example.

- Estimation Performances

In [3], Hsieh et al. has proved that the OTSKF is equivalent to the ASKF, so the tracking results of the two algorithms are the same. In order to make the figure clearer, we will only compare the performances of the following six algorithms:

Algorithm 1: KF without considering unknown input.

Algorithm 2: ASKF with accurate initial value of unknown input $\left(d_{0}={\left(5,5\right)}^{T}\right)$.

Algorithm 3: OTSKF with accurate initial value of unknown input $\left(d_{0}={\left(5,5\right)}^{T}\right)$.

Algorithm 4: ASKF with wrong inaccurate initial value of unknown input $\left(d_{0}={\left(0,0\right)}^{T}\right)$.

Algorithm 5: ASKF with inaccurate initial value of unknown input $\left(d_{0}={\left(20,20\right)}^{T}\right)$.

Algorithm 6: Difference KF without any information about initial value of unknown input.

The initial states of the six algorithms are set at $x_{0|0}=x_{0}$, the initial $P_{x_{0|0}}=R_{v_{0}}$, $P_{d_{0|0}}=R_{d_{0}}$. Using 100 Monte-Carlo runs, we can evaluate estimation performance of an algorithm by estimating the second moment of the tracking error:

$$E_{k}^{2}=\frac{1}{100}\sum_{j=1}^{100}||x_{k|k}^{\left(j\right)}-x_{k}|{|}^{2},\;k=1,2,\cdots ,100.$$

**Figure 1:**
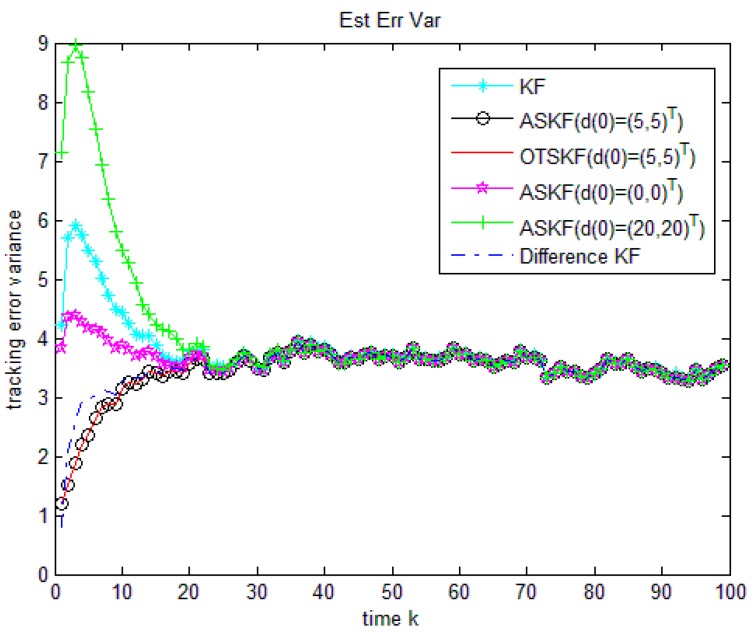
Comparison of the six algorithms when $d_{k}$ is a stationary time series.

It must be noticed that Difference KF uses $\left(y_{1},y_{2},\cdots ,y_{k},y_{k+1}\right)$ to estimate $x_{k}$ at step k. However, the KF, ASKF and OTSKF only use $\left(y_{1},y_{2},\cdots ,y_{k}\right)$ to estimate $x_{k}$ at step k. To make an equal comparison, $x_{k|k-1}$ in Difference KF with $x_{k|k}$ in the other five algorithms is compared. As $d_{k+1}=0.9d_{k}+\omega_{k}$, $d_{k}$ is almost equal to a random white noise with small covariance after several steps and the influence of the initial value $d_{0}$ will be gradually weakened. The tracking errors of the six methods are compared in Figure 1 and Table 2. It can be noticed that no matter whether the initial values of the unknown input in ASKF and OTSKF are accurate or wrong, the tracking results of the six algorithms are almost the same after about 25 steps. However, it should be noticed that the Difference KF performs better than the ASKF with inaccurate initial value of unknown measurement in the first stage, which is important for some practical conditions, for instance, in multi-target tracking problems, due to data association errors and heavy clutters, tracking has to restart very often. Therefore, in order to derive an entirely good tracking, initial work status at each tracking restarting should be as good as possible.

**Example** **2.** *The dynamic equations are the same as Example 1. Assume* $B_{k}=I$ *. This model has been considered in [4,5].* $d_{k}$ *is a non-stationary time series here. The non-stationary unknown measurement is common in practice. For instance, for an air target, the unknown radar bias is frequently increasing with distance changing between the target and radar.*

The targets start at $x_{0}={\left(50,1,50,1\right)}^{T}$ and the initial value $d_{0}={\left(5,5\right)}^{T}$.The performances of the following six algorithms are compared:

Algorithm 1: KF without considering unknown input.

Algorithm 2: ASKF with accurate initial value of unknown input $\left(d_{0}={\left(5,5\right)}^{T}\right)$.

Algorithm 3: OTSKF with accurate initial value of unknown input $\left(d_{0}={\left(5,5\right)}^{T}\right)$.

Algorithm 4: ASKF with wrong inaccurate initial value of unknown input $\left(d_{0}={\left(0,0\right)}^{T}\right)$.

Algorithm 5: ASKF with inaccurate initial value of unknown input $\left(d_{0}={\left(20,20\right)}^{T}\right)$.

Algorithm 6: Difference KF without any information about initial value of unknown input.

**Figure 2:**
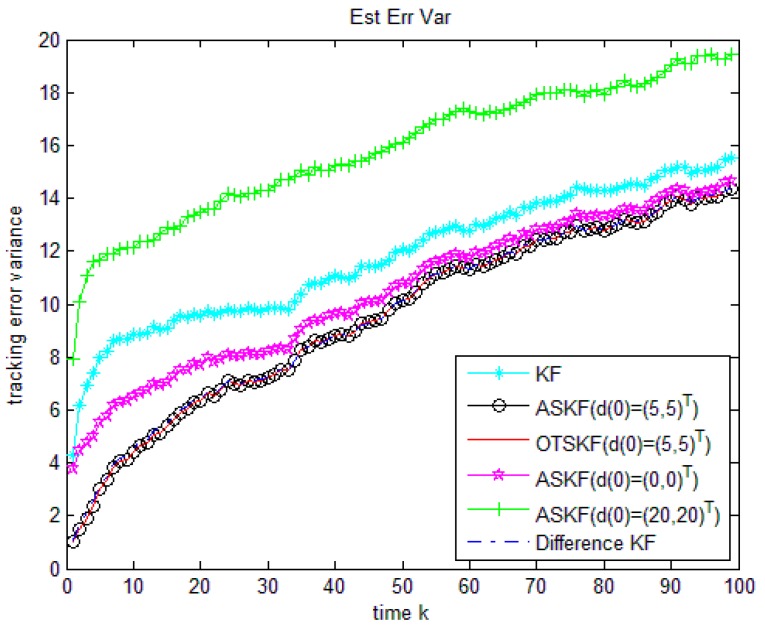
Comparison of the six algorithms when $d_{k}$ is a non-stationary time series.

Figure 2 and Table 3 compare the tracking errors of the six methods. As the new algorithm, ASKF and OTSKF with accurate initial value of unknown input are optimal in the MSE sense. Their performances are almost of no difference. The KF without considering unknown input is worse because it does not use any information of the unknown input. Numerical examples also demonstrate that once the initial value of the unknown input is inaccurate, the performance of the ASKF becomes poorer. We can also see that if the initial value of the unknown input is largely biased, the performance of ASKF is even poorer than KF ignoring unknown input. This is because $d_{k+1}=d_{k}+\omega_{k}$ in this example, the influence of the incorrect initial value $d_{0}$ will always exist. Nevertheless, the new algorithm is independent of the initial value of the unknown input and yet performs well.

From Examples 1 and 2, we can see that the performance of the difference KF is almost the same to that of the ASKF and OTSKF with accurate initial value of unknown input. If the initial value of the unknown measurement is largely biased, performances of the ASKF and OTSKF will be badly influenced. Due to the fact that it is not easy to get the exact initial value of the unknown measurement, Difference KF is a better option than the ASKF and OTSKF in practice.

**Example** **3.** *A two-sensor Kalman filtering fusion problem with unknown inputs is considered. The object dynamics and measurement equation are modeled as follows:*

$$x_{k+1}=F_{k}x_{k}+\nu_{k},\;k=0,1,\ldots ,100$$

$$y_{k}^{i}=H_{k}^{i}x_{k}+A_{k}^{i}d_{k}^{i}+\omega_{k}^{i},$$

$$d_{k+1}^{i}=B_{k}^{i}d_{k}^{i}+\omega_{d_{k}}^{i},\;i=1,2.$$

*The state transition matrix* $F_{k}$ *and the measurement matrices* $H_{k}^{i}$ *are the same as Example 1,* $A_{k}^{i}$ *and* $B_{k}^{i}$ *are identity matrix with appropriate dimensions. The targets start at* $x_{0}={\left(50,1,50,1\right)}^{T}$ *and the initial value* $d_{0}^{i}={\left(5,5\right)}^{T}$ *.*

*The covariance matrices of the process noises is given by:*

$$R_{\nu_{k}}=\left(\begin{array}{cccc} 5 & 0 & 0 & 0\\ 0 & 0.1 & 0 & 0\\ 0 & 0 & 5 & 0\\ 0 & 0 & 0 & 0.1 \end{array}\right)$$

*The covariance matrices of the measurement noises and the unknown inputs are diagonal given by* $R_{\omega_{k}}^{i}=1,R_{d_{k}}^{i}=1,i=1,2$ *.*

The performances of the following three algorithms are compared as follows:

Algorithm 1: Centralized KF without considering unknown input.

Algorithm 2: The centralized fusion of the Difference KF.

Algorithm 3: The distributed fusion of the Difference KF.

The initial states of the three algorithms are set at $x_{0|0}=x_{0}$, the initial $P_{x_{0|0}}^{i}=I$. Using 100 Monte-Carlo runs, we can evaluate estimation performance of an algorithm by estimating the second moment of the tracking error.

**Figure 3:**
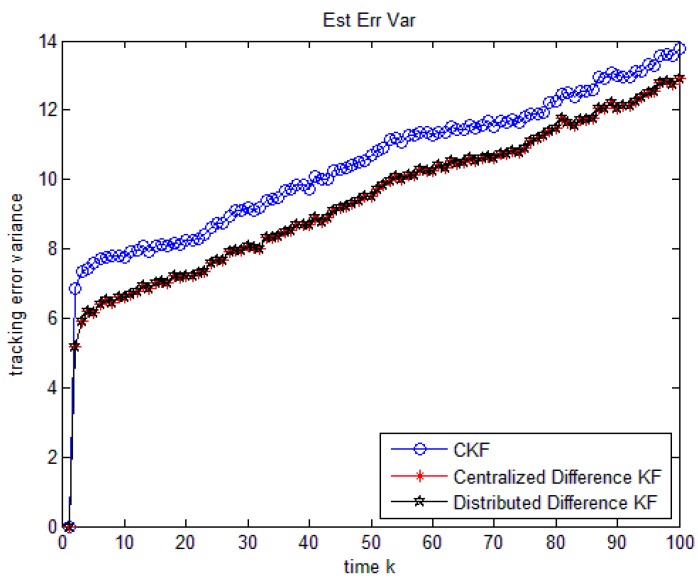
Comparison of the three algorithms in multisensor case.

It is illustrated in Figure 3 and Table 4 that the simulation outcome of distributed fusion and centralized fusion of the new algorithm are exactly the same. Additionally, the new algorithm fusion gives better performance than the KF. Thus, the distributed algorithm has not only the global optimality, but also the good survivability in a poor situation.

## 6. Conclusions

In this paper, the state estimation for dynamic system with unknown inputs modeled as an autoregressive AR (1) process is considered. The main contributions are: (1) A novel optimal algorithm for dynamic system with unknown inputs in the MSE sense is proposed by differential method. The computational burden of the new algorithm is lower than that of ASKF. The performance of the new algorithm is independent of the initial value of the unknown input. (2) A distributed fusion algorithm is proposed for the multisensor dynamic system with unknown inputs, the result of which is equal to the centralized difference Kalman filtering adopting all sensor measurements. 

However, it should be noticed that the new algorithm uses $y_{k}$ and $y_{k+1}$ to estimate $x_{k}$, which leads the new algorithm to be one-step delayed. The new algorithm can only cope with the unknown inputs in measurement equation, while the ASKF can handle the unknown inputs in both state and measurement equation. Besides, it is assumed throughout the paper that the model of the unknown inputs $d_{k}$ follows an autoregressive AR (1) process. As for the future research, one interesting direction is to extend the difference method to dynamic system with more general unknown inputs.

## Figures and Tables

**Table 1:** The computer time of the three algorithms.

Algorithm	Computer Time (seconds)
ASKF	16.163026
OTSKF	11.684104
Difference KF	9.274128

**Table 2:** The average tracking errors of the six methods.

**Algorithm**	KF	ASKF $d_{0}={\left(5,5\right)}^{T}$	OTSKF $d_{0}={\left(5,5\right)}^{T}$	ASKF $d_{0}={\left(0,0\right)}^{T}$	ASKF $d_{0}={\left(20,20\right)}^{T}$	Difference KF
**Average Tracking Error**	3.6843	3.3261	3.3261	3.5335	3.9229	3.3540

**Table 3:** The average tracking errors of the six methods.

**Algorithm**	KF	ASKF $d_{0}={\left(5,5\right)}^{T}$	OTSKF $d_{0}={\left(5,5\right)}^{T}$	ASKF $d_{0}={\left(0,0\right)}^{T}$	ASKF $d_{0}={\left(20,20\right)}^{T}$	Difference KF
**Average Tracking Error**	11.8091	9.5470	9.5470	10.4493	15.8539	9.5787

**Table 4:** The average tracking errors of the three methods.

**Algorithm**	CKF	Centralized Difference KF	Distributed Difference KF
**A** **verage Tracking Error**	10.3720	9.3582	9.3582
